# Age and Birth Cohort–Adjusted Rates of Suicide Mortality Among US Male and Female Youths Aged 10 to 19 Years From 1999 to 2017

**DOI:** 10.1001/jamanetworkopen.2019.11383

**Published:** 2019-09-13

**Authors:** Bin Yu, Xinguang Chen

**Affiliations:** 1 Department of Epidemiology, University of Florida, Gainesville

## Abstract

**Importance:**

Strategic planning to curb increasing suicide rate among US youths requires unbiased measures of suicide mortality, as the unadjusted suicide mortality rates conventionally used in describing time trends and sex patterns are confounded by the differences in chronological age and year of birth.

**Objective:**

To measure time trends and sex differences in suicide mortality among US youths using age and birth cohort (age-cohort)–adjusted rates.

**Design, Setting, and Participants:**

This population-based multiyear cross-sectional study of US youths aged 10 to 19 years was a secondary analysis of the suicide data for January 1, 1999, to December 31, 2017, derived from the Wide-Ranging Online Data for Epidemiologic Research, a database prepared by the US Centers for Disease Control and Prevention to promote research. Data analysis was conducted from May 24 to May 27, 2019.

**Main Outcomes and Measures:**

Age-cohort–adjusted suicide mortality rates by year and sex were used as the main outcomes. These adjusted rates were calculated based on the period effect estimated with the age-period-cohort (APC) modeling method. Participants’ age, year of birth, and the year when suicide occurred were used as time-related risk factors.

**Results:**

Age-cohort–adjusted suicide mortality rates were estimated successfully by year and by sex. The adjusted rates were obtained after controlling for a V-shaped cohort effect and a curved age effect that also differed by sex. The unadjusted suicide mortality rate for male youths increased from 7.4 per 100 000 in 1999 to 10.7 per 100 000 in 2017, while the adjusted rate increased from 4.9 per 100 000 in 1999 to 8.7 per 100 000 in 2017. The unadjusted suicide mortality rates for female youths were 1.6 per 100 000 in 1999 and 3.5 per 100 000 in 2017, while the adjusted rates were 1.7 per 100 000 in 1999 and 4.2 per 100 000 in 2017.

**Conclusions and Relevance:**

Findings of this study indicate a more rapid increase and smaller male-female difference in suicide rates among US youths since 1999 than those shown by the unadjusted suicide mortality rates. The adjusted rates provide unbiased data that are needed for evidence-based decision-making and strategies to curb the increasing suicide rates among US youths.

## Key Points

**Question:**

Does a measure of suicide mortality calculated by adjusting for chronological age and year of birth differ from unadjusted models?

**Findings:**

In this multiyear cross-sectional study using data from the US Centers for Disease Control and Prevention Wide-Ranging Online Data for Epidemiologic Research database, age and birth cohort–adjusted suicide mortality rates were estimated after controlling for a curved age effect and a V-shaped cohort effect that also differed by sex. The adjusted rates showed a more rapid increase and a smaller sex difference in the suicide mortality rates for US youths aged 10 to 19 years from 1999 to 2017.

**Meaning:**

Age and birth cohort–adjusted suicide mortality rates may be an unbiased measure of the time trend and sex pattern in suicide among US youths and may be useful for informing evidence-based suicide prevention.

## Teaser

This cross-sectional study examines time trends and sex differences in the suicide mortality rate among US youths adjusted for birth cohort and age.

## Introduction

Valid measures are essential for evidence-based planning and decision-making in suicide prevention to control the increasing trends in suicide among youths in the United States.^1,2^ The suicide mortality rate, or number of suicide deaths per 100 000 individuals, has been used in describing the epidemic of suicide.^1,3^ Based on the unadjusted rate in the past 2 decades, suicide among US youths showed a higher and V-shaped trend for male youths and a lower and relatively smooth trend of increase for female youths, with a declining sex difference.^3,4,5^

Despite their wide acceptance and frequent use in research and practice, the reported suicide mortality rates are very likely to be biased if used to describe time trends and sex differences.^6,7,8,9,10^ This is because the suicide mortality rate for a given year consists of 3 time-related components: chronological age, birth year (also known as *birth cohort*), and period when suicide occurred. Studies using the age-period-cohort (APC) modeling method have demonstrated the existence of the 3 components through empirical data.^7,8,11,12,13,14^ Methods to obtain an age-adjusted rate have been well established for bias correction^9^; however, an age-standardized rate remains confounded by potential differences in the mortality of people who were born in different years. In this study, we applied a new approach to obtain age and birth cohort (age-cohort)–adjusted suicide mortality rates, capitalizing on a classic epidemiologic method of APC modeling capable of controlling the impacts of both the age and cohort.

**Figure 1.**
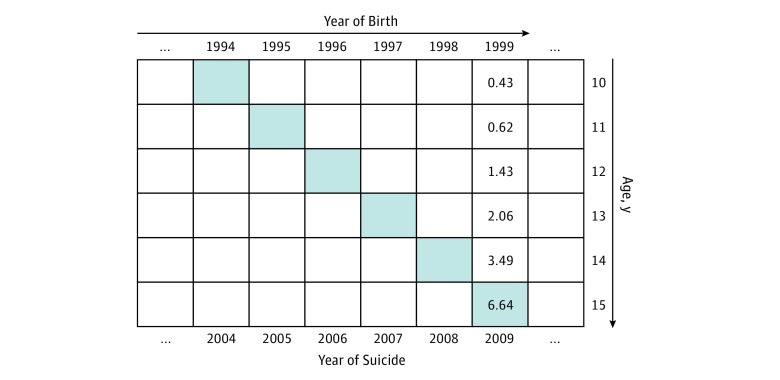
Schematic Illustration of the Age, Period, and Birth Cohort Effects With Hypothetical Data In this example, the suicide mortality rate of 6.64 per 100 000 for individuals aged 15 years consists of 3 components: 1 contributing to the mortality rate in 2009, 1 attributing to age from birth to age 15 years, and 1 attributing to the birth year of 1994 (following the shaded boxes from top left to bottom right).

To illustrate the new method, we used the suicide mortality rate for youths aged 10 to 15 years in 2009 as an example using hypothetical data (Figure 1). Epidemiologically, the recorded suicide mortality rate of these youths in 2009 consists of 3 independent components: (1) suicide risk associated with the chronological age of these youths by age from ages 10 years to 15 years in 2009, and the variations in the suicide risk by age, also known as *age effect*, which will contribute to the recorded suicide mortality in 2009; (2) suicide risk by birth cohort for youths born in 1994 (15 years ago) to 1999 (10 years ago), and variations in the suicide risk by birth cohort, also known as *cohort effect*, which will also contribute to the recorded suicide mortality in 2009; and (3) suicide risk in 2009 when the recorded suicide deaths occurred, also known as *period effect*.^11^

In Figure 1, assuming 2.45 persons per 100 000 completed suicide in 2009, this rate does not correctly reflect the level of suicide in 2009 (the period effect) unless the impacts of age and year of birth are adjusted for. This is because the rate of 2.45 per 100 000 in 2009 contains 2 more components: (1) differences in the risk of suicide by age from 10 to 15 that are contained in the age-specific mortality varying from 0.43 to 6.64 per 100 000 (ie, age effect); (2) differences in the risk of suicide by years of birth from 1994 to 1999 (ie, cohort effect) that are also contained in the age-specific rates. Therefore, using age-specific suicide mortality rates as data, unbiased measure of suicide mortality rates can be obtained based on the period effect. The purpose of this study is to obtain unbiased measures of suicide mortality rates to describe time trend and sex difference with the period effect derived from the age-specific rate using APC modeling method to adjust for age and cohort effects.

## Methods

### Data Source

Deidentified suicide data were extracted from the Wide-Ranging Online Data for Epidemiologic Research database,^15^ a public domain database prepared by the US Centers for Disease Control and Prevention to promote research. Total population and suicide deaths by single year of age from 10 to 19 years and by sex from 1999 to 2017 were included. Suicide deaths were those coded as *U03*, *X60-X84*, and *Y87.0* in the Wide-Ranging Online Data for Epidemiologic Research database, following the definition of suicide in the *International Statistical Classification of Diseases and Related Health Problems, Tenth Revision*.^16^ This study was guided by Strengthening the Reporting of Observational Studies in Epidemiology (STROBE) reporting guideline for cross-sectional studies and was approved by the institutional review board at the University of Florida. Informed consent was waived because the data were deidentified.

### Time-Related Risk Factors

The 3 examined time-related risk factors were age in years, year of birth, and year when a suicide occurred. The registered age of suicide death was used as the variable age, and it ranged from 10 to 19 years. The year when the reported suicide death occurred was used as the variable period, and it ranged from 1999 to 2017. The year of birth was computed by subtracting the age from the period and was used as the variable cohort; it ranged from 1980 to 2007. These 3 time-related variables were used for APC modeling analysis.

### Statistical Analysis

Annual suicide mortality rates per 100 000 individuals by single year of age and sex were computed as the number of suicide deaths divided by population estimates for 1999 through 2017. These computed suicide mortality rates were analyzed using the APC modeling approach, assuming that the occurrence of suicide deaths follows a Poisson distribution.^10,11^

Using *r_ijk_* as the estimated suicide mortality rate for youth in age group *i*, time period *j*, and birth cohort *k*, the following log-linear regression was used for APC modeling in Equation 1:

log(*r_ijk_*) = *u* + *α_i_*(age*_i_*) + *β_j_*(period*_j_*) + *γ_k_*(cohort*_k_*),

in which *u* was the intercept or grand mean, *α_i_* was the effect for age group *i* (eg, *i* = 10, 11, 12), *β_j_* was the effect for period *j* (eg, *j* = 1999, 2000, 2001), and *γ_k_* was the effect for birth cohort *k* (eg, *k* = 1980, 1981, 1982).

The model parameters in Equation 1 were estimated using the intrinsic estimator.^10,11^ The modeling analysis was implemented using Stata statistical software version 15.0 with a special software package apc_ie (StataCorp). Thus, the estimated age, period, and cohort effects were manually plotted by sex to visualize changes in the risk pattern of suicide along with the 3 time-related risk variables.^7^

Finally, the age-cohort–adjusted suicide mortality rate was estimated based on the period effect that was independent of the chronological age and birth cohort using Equation 2: 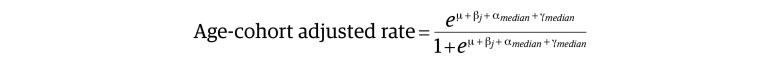
in which *u* was the intercept, *β_j_* was the effect for period *j* (eg, *j* = 1999, 2000, 2001), α*_median_* was the median of age effects, and γ*_median_* was the median of cohort effects, all estimated through the APC modeling analysis as described by Equation 1. Computation of the age-cohort–adjusted rates was completed using an Excel spreadsheet (Microsoft Corp). The adjusted rates by year were plotted together with the unadjusted rates for male youths and female youths to reveal the differences for efficient result interpretation.

Unlike most reported studies to our knowledge, data for the total population of US youths were analyzed in this study. Consequently, no type I error due to sampling was involved; therefore no significance test was conducted for statistical analyses.

## Results

### Suicide Mortality Rates for US Youths

The Table presents the unadjusted suicide mortality rates by year of age and by year for male and female youths. Suicide mortality among US youths increased continuously from 2007 after a prior trend of decline since 1999. This trend appears to be consistent by age and sex. Additionally, the data in the Table show 2 distinctive patterns: (1) suicide mortality rates increased with age in all periods and (2) suicide mortality rates were much higher for male youths than for female youths.

**Table.** Age-Specific Rate of Suicide Death per 100 000 US Youths Aged 10 to 19 Years^a^

Age, y	Suicide Mortality Rate per 100 000 Youths by Year																		
	1999	2000	2001	2002	2003	2004	2005	2006	2007	2008	2009	2010	2011	2012	2013	2014	2015	2016	2017
**Male**																			
10	0.3	0.6	0.1	0.7	0.4	0.5	0.3	0.1	0.5	0.1	0.4	0.2	0.4	0.4	0.2	0.4	0.6	0.4	0.5
11	1.0	1.0	0.9	0.9	0.6	0.6	1.1	0.9	0.6	0.5	0.6	0.7	0.7	0.4	0.6	1.3	1.0	1.1	1.5
12	1.1	1.7	1.5	1.4	1.4	1.2	1.4	1.0	1.1	0.9	1.4	1.2	1.4	1.7	1.9	2.0	1.5	2.0	2.4
13	2.3	3.7	3.0	2.6	2.4	2.5	2.1	1.9	1.5	2.3	2.1	2.2	2.6	3.0	3.3	3.4	3.1	3.3	4.2
14	4.7	4.5	4.2	3.5	3.9	3.6	4.3	3.0	2.3	3.2	3.5	4.2	4.2	4.9	5.5	5.7	5.8	5.8	8.0
15	5.4	7.1	5.7	5.9	5.6	5.4	5.8	5.9	5.3	5.1	6.6	5.6	6.6	7.6	7.7	7.5	7.7	10.0	11.8
16	10.5	10.5	9.2	9.4	8.0	9.4	7.8	7.7	7.6	8.2	8.7	8.8	10.0	8.7	9.7	10.6	10.5	11.4	14.7
17	13.8	12.4	13.5	11.2	10.8	11.0	12.0	10.7	9.7	11.7	11.2	10.7	12.2	12.2	12.0	13.1	14.8	14.2	16.5
18	17.7	16.8	15.5	14.8	14.1	16.6	15.4	13.8	14.7	13.1	15.0	16.1	15.8	15.1	14.4	16.3	16.5	18.3	20.6
19	17.7	18.0	19.8	18.9	18.6	19.5	18.3	18.6	16.6	18.0	16.2	16.8	19.2	18.4	18.1	17.6	21.6	20.2	25.9
Total	7.4	7.6	7.3	6.9	6.5	7.0	6.9	6.4	6.1	6.5	6.8	6.9	7.5	7.4	7.5	7.9	8.4	8.8	10.7
**Female**																			
10	0	0.1	0	0	0	0.1	0.1	0	0.1	0.1	0.1	0.1	0.1	0.1	0.2	0	0.2	0.2	0.1
11	0.1	0.1	0.2	0.1	0.1	0.3	0.2	0.1	0.3	0.2	0.2	0.3	0.1	0.4	0.5	0.5	0.3	0.5	0.5
12	0.5	0.4	0.3	0.6	0.4	0.5	0.4	0.4	0.1	0.3	0.9	0.6	0.6	0.4	1.2	1.3	1.2	1.3	1.3
13	0.4	1.0	1.1	1.0	0.7	1.6	0.9	0.8	0.8	0.7	1.5	1.5	1.2	0.9	2.2	2.9	2.7	2.5	3.0
14	1.5	1.6	1.7	1.4	1.5	2.2	1.7	1.9	1.3	2.0	1.6	1.8	2.2	2.4	3.0	2.7	3.4	4.0	3.4
15	2.7	1.9	2.5	2.1	2.1	2.8	2.5	2.7	1.9	2.9	2.8	2.6	3.3	4.1	3.5	4.2	4.1	4.6	5.1
16	2.5	3.4	2.7	2.1	3.0	3.2	2.5	2.7	2.5	3.5	3.3	3.4	3.5	4.0	3.9	4.3	5.1	5.8	5.9
17	2.3	3.0	2.6	3.3	2.1	3.1	3.5	2.6	1.9	2.8	3.3	2.4	3.6	3.8	3.8	3.9	4.3	5.3	5.1
18	2.5	2.7	2.2	2.3	3.0	4.0	2.7	2.4	2.8	2.2	2.8	3.4	3.8	3.9	3.8	4.3	5.6	4.6	5.5
19	3.7	2.7	3.4	2.0	2.9	4.2	3.7	3.4	3.0	3.4	3.7	3.7	3.5	4.0	4.3	4.2	6.3	4.8	5.3
Total	1.6	1.7	1.6	1.5	1.6	2.2	1.8	1.7	1.5	1.9	2.1	2.0	2.2	2.4	2.6	2.8	3.3	3.4	3.5

^a^ From the US Centers for Disease Control and Prevention Wide-Ranging Online Data for Epidemiologic Research database.^15^

### Age, Period, and Cohort Effects From APC Modeling

**Figure 2.**
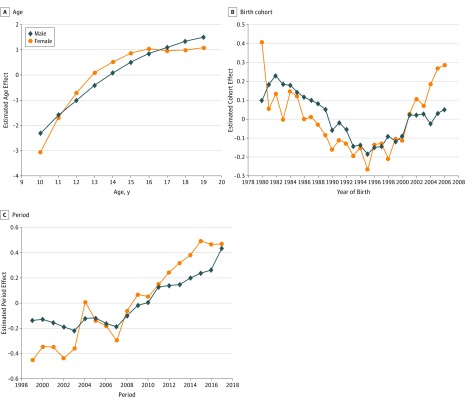
Age-Period-Cohort Modeling Estimated Effect Size for US Youths by Sex

The age effect derived from APC modeling analysis indicated a curve-linear association of chronological age with suicide risk, with the effect for female youths increasing more rapidly than for male youths from ages 10 to 16 years (Figure 2A). Likewise, Figure 2B shows a V-shaped pattern of birth cohort effect on suicide mortality for US youths born since 1980, with the lowest effect for those born in 1995. Furthermore, compared with male youths, the cohort effect for female youths fluctuated more dramatically, with a lower level for those born before 1995 and a higher level for those born after 2001. To obtain a valid measure of the time trend and sex differences, these large and varying effects from chronological age-cohort must be considered.

The estimated period effect (Figure 2C) reflected the underlying suicide risk during 1999 to 2017 that was independent from the age and cohort. Relative to male youths, the suicide risk for female youths started at a lower level, increased more rapidly, and overtook that of male youths after 2007, with bigger fluctuations in the periods from 2001 to 2007. Because the estimated period effects were independent of age and cohort, they were used to estimate age-cohort–adjusted suicide mortality rates.

### Age-Cohort–Adjusted Suicide Mortality Rate

**Figure 3.**
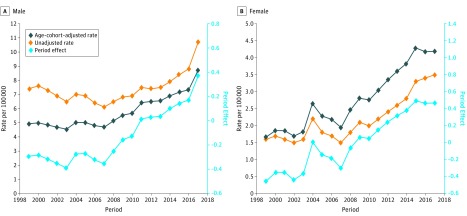
Age-Cohort–Adjusted and Unadjusted Suicide Mortality Rate and Age-Period-Cohort Model Estimated Period Effect Size of Suicides for US Youths Aged 10 to 19 Years From 1999 to 2017

Age-cohort–adjusted suicide mortality rates were calculated based on the estimated period effect (Figure 2C). The adjusted rates were presented together with the unadjusted rates, and the estimated period effect from APC modeling for male youths (Figure 3A) and female youths (Figure 3B). Compared with the unadjusted rates, the adjusted rates were not affected by the chronological age or year of birth and reflected the real-time trends and sex differences.

For male youths, the adjusted rates were lower than the unadjusted rates across the period, with a larger difference before 2008 corresponding with the 1981 through 1998 birth cohorts, when these youths were born, and with a higher cohort effect for male youths than for female youths (Figure 2B). Further, according to the adjusted rates, there were no declines in the suicide mortality rates before 2007, contrary to what is suggested by the unadjusted suicide mortality rates.

In contrast to male youths, the adjusted suicide mortality rates for female youths were higher than the unadjusted rates, with a larger difference in more recent years and a more rapid increase over time compared with the unadjusted rates. According to the adjusted rates, the unadjusted rates underestimated the level and time trend in suicide mortality for US female youths, since the unadjusted rates were confounded by the curved age effect (Figure 2A) and V-shaped cohort effect (Figure 2B), particularly the lower cohort effect before 2001 for female vs male youths.

**Figure 4.**
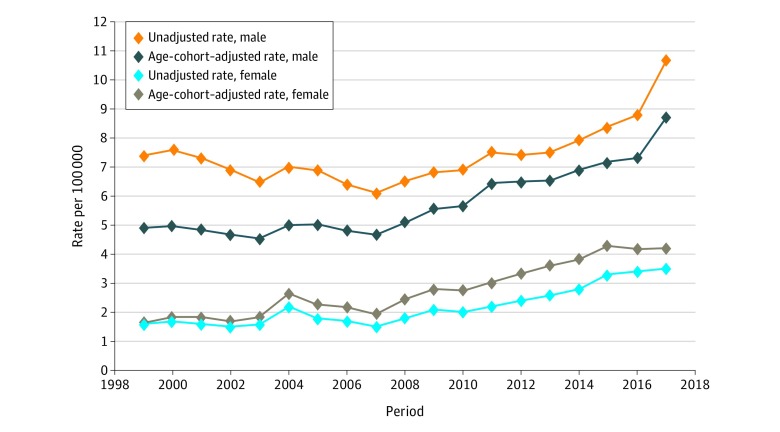
Male-Female Differences in the Age-Cohort–Adjusted and Unadjusted Suicide Mortality Rates per 100 000 US Youths Aged 10 to 19 Years

Figure 4 further contrasts the sex differences in the adjusted and unadjusted suicide mortality rates during the period. The unadjusted suicide mortality rate for male youths increased from 7.4 per 100 000 in 1999 to 10.7 per 100 000 in 2017, while the adjusted rate increased from 4.9 per 100 000 in 1999 to 8.7 per 100 000 in 2017. The unadjusted suicide mortality rates for female youths were 1.6 per 100 000 in 1999 and 3.5 per 100 000 in 2017, while the adjusted rates were 1.7 per 100 000 in 1999 and 4.2 per 100 000 in 2017. According to the adjusted rates, there was no significant decline in suicide mortality for male youths during 1999 to 2007 as suggested by the unadjusted rates. Additionally, the unadjusted rates overestimated suicide mortality for male youths in early periods but underestimated suicide mortality for female youths in more recent periods, with the overall sex difference being overestimated. For example, the unadjusted rates present the sex differences in suicide deaths as 5.8 per 100 000 in 1999 and 7.2 per 100 000 in 2017, whereas the age-cohort–adjusted rates reduce those differences to 3.2 per 100 000 in 1999 and 4.5 per 100 000 in 2017.

## Discussion

In this study, we calculated the age-cohort–adjusted suicide mortality rates to describe the time trends and sex difference in suicide mortality among US youths aged 10 to 19 years from 1999 to 2017. The adjusted rates were calculated based on the period effect estimated using the classic epidemiologic method of APC modeling to control for the effect of chronological age and birth year. The adjusted rates provided unbiased data for evidence-based planning and decision-making for suicide prevention among US youths.

The age-cohort–adjusted suicide mortality rates indicated that suicide mortality among US youths increased continuously and more rapidly than the unadjusted rates had suggested. The unadjusted rates for male youths showed a declining trend before 2007, but this trend disappeared when measured by adjusted rates. The adjusted suicide mortality rate increased more rapidly than the unadjusted rate among female youths. We found that the differences between the adjusted and the unadjusted rates were primarily due to the effect of chronological age and birth year. Although the increasing trend in suicide among youths has been reported in the literature,^1,4,5^ findings from the adjusted rates indicate that even more urgent action is needed to control the increasing trend.

Although a systematic examination of factors associated with the increasing trends is beyond the scope of this study, a short-term increase in adjusted suicide risk during 2003 to 2005 could be due to reductions in the prescribed antidepressants in response to the regulation issued by the US Food and Drug Administration in 2004.^17^ The progressive increases in the adjusted suicide mortality rates for both sexes during the period was associated with the increases in social media exposure^18^ and substance abuse.^19^ Additional studies with new data are needed to examine factors associated with the increasing trends in suicide.

The sex difference measured by adjusted rates was smaller than that of the unadjusted rate for US youths during the period. Measured by the unadjusted rates, the male-female differences in the rate of suicide deaths youths was 5.8 per 100 000 in 1999 and 7.2 per 100 000 in 2017; however, the sex differences in the 2 years were reduced to 3.2 per 100 000 in 1999 and 4.5 per 100 000 in 2017 when the adjusted rates were used. This finding suggests that we cannot ignore the risk of suicide among female youths, as their risk of suicide was higher than the unadjusted rates shown in this study and those of others in the literature.^3,5,20^

In addition to the adjusted rates, this study generated 2 by-products: age effect and cohort effect. First, consistent with published data,^12,13^ there was a curve-linear association between age and the risk of suicide, with the risk increasing with age from 10 to 19 years old. This finding suggests the importance of early prevention to reduce the suicide risk among youths in the United States. Furthermore, the risk started at a lower level and increased more rapidly for female youths than for male youths up to age 16 years. This sex difference could be associated with the earlier emotional and cognitive maturation of female youths compared with male youths.^21,22^ Further studies are needed to investigate the difference in risk associated with age. Suicide prevention guidelines and strategies should also consider this sex difference.

This study revealed a V-shaped cohort effect with a decline in suicide mortality risk for those born from 1980 to 1995 and an increase in suicide mortality risk for those born after 1995. The increased cohort effect of suicide mortality risk after 1995 corresponded with the later Millennial Generation and Generation Z, characterized by long-term internet use and intense social media exposure, both of which have previously been associated with increased risk of suicide.^18,23,24^

### Limitations

This study has limitations. First, APC modeling uses aggregated data to separate the period effect from the other 2 time-related variables of chronological age and birth year. Therefore, it cannot examine factors associated with the time trend using individual-level data. Second, this study used *International Statistical Classification of Diseases and Related Health Problems, Tenth Revision*^16^ codes to classify suicide death; misclassification cannot be completely ruled out. Despite the limitations, findings of this study provide new data to measure time trends and sex differences in suicide mortality among US youths. These data are needed for evidence-based planning and precision intervention to reduce the suicide rate.

## Conclusions

To our knowledge, this is the first study to apply the age-cohort–adjusted rates in describing the occurrence of suicide among US youths. Compared with unadjusted suicide mortality rates, the age-cohort–adjusted rates indicate a more rapid increase in suicide mortality among US youths during 1999 to 2017. Compared with the unadjusted suicide mortality rates, the adjusted suicide mortality rates were lower for male youths and higher for female youths, and the sex differences were smaller. This study suggests that there is a need for greater efforts to control the rapidly increasing suicide mortality trend, with extra attention given to female youths.
